# Increased Susceptibility of Humanized NSG Mice to Panton-Valentine Leukocidin and *Staphylococcus aureus* Skin Infection

**DOI:** 10.1371/journal.ppat.1005292

**Published:** 2015-11-30

**Authors:** Ching Wen Tseng, Juan Carlos Biancotti, Bethany L. Berg, David Gate, Stacey L. Kolar, Sabrina Müller, Maria D. Rodriguez, Kavon Rezai-Zadeh, Xuemo Fan, David O. Beenhouwer, Terrence Town, George Y. Liu

**Affiliations:** 1 Division of Pediatric Infectious Diseases and the Immunobiology Research Institute, Cedars-Sinai Medical Center, Los Angeles, California, United States of America; 2 Department of Pediatrics, David Geffen School of Medicine, University of California, Los Angeles, California, United States of America; 3 Zilkha Neurogenetic Institute, Department of Physiology and Biophysics, Keck School of Medicine, University of Southern California, Los Angeles, California, United States of America; 4 Department of Biomedical Sciences, Cedars-Sinai Medical Center, Los Angeles, California, United States of America; 5 Department of Pathology and Laboratory Medicine, Cedars-Sinai Medical Center, Los Angeles, California, United States of America; 6 Division of Infectious Diseases, Veterans Affairs Greater Los Angeles Healthcare System, Los Angeles, California; Department of Medicine, David Geffen School of Medicine, University of California, Los Angeles, California, United States of America; 7 Department of Medicine, David Geffen School of Medicine, University of California, Los Angeles, California, United States of America; Trinity College Dublin, IRELAND

## Abstract

*Staphylococcus aureus* is a leading cause of skin and soft-tissue infections worldwide. Mice are the most commonly used animals for modeling human staphylococcal infections. However a supra-physiologic *S*. *aureus* inoculum is required to establish gross murine skin pathology. Moreover, many staphylococcal factors, including Panton-Valentine leukocidin (PVL) elaborated by community-associated methicillin-resistant *S*. *aureus* (CA-MRSA), exhibit selective human tropism and cannot be adequately studied in mice. To overcome these deficiencies, we investigated *S*. *aureus* infection in non-obese diabetic (NOD)/severe combined immune deficiency (SCID)/IL2rγ^null^ (NSG) mice engrafted with human CD34^+^ umbilical cord blood cells. These “humanized” NSG mice require one to two log lower inoculum to induce consistent skin lesions compared with control mice, and exhibit larger cutaneous lesions upon infection with PVL^+^ versus isogenic PVL^-^ *S*. *aureus*. Neutrophils appear important for PVL pathology as adoptive transfer of human neutrophils alone to NSG mice was sufficient to induce dermonecrosis following challenge with PVL^+^ *S*. *aureus* but not PVL^-^ *S*. *aureus*. PMX53, a human C5aR inhibitor, blocked PVL-induced cellular cytotoxicity *in vitro* and reduced the size difference of lesions induced by the PVL^+^ and PVL^-^ *S*. *aureus*, but PMX53 also reduced recruitment of neutrophils and exacerbated the infection. Overall, our findings establish humanized mice as an important translational tool for the study of *S*. *aureus* infection and provide strong evidence that PVL is a human virulence factor.

## Author Summary

*S*. *aureus* infection has emerged in the past decade as a major burden to public health and is responsible for a surge in preclinical research. Mice are the most commonly studied animals for modeling of human *S*. *aureus* infection. However, it is increasingly evident that available murine models poorly mimic human *S*. *aureus* disease. Routinely, a supra-physiologic inoculum is required to establish soft-tissue pathology. Additionally, many *S*. *aureus* factors exhibit unique human tropism and cannot be adequately investigated in rodents. Here we investigated *S*. *aureus* infection in NSG mice engrafted with human umbilical CD34^+^ cells. We showed that a one to two log lower infectious inoculum of *S*. *aureus* establishes consistent skin lesions in humanized NSG mice. This inoculum is comparable to published inocula required to induce infection in humans. In addition, we showed that Panton-Valentine Leucocidin, a human tropic factor secreted by *S*. *aureus*, contributes to the development of dermonecrosis in the humanized mice, and its interaction with human neutrophils and human C5a receptor appears to be important for immunopathology. Overall our study suggests that humanized mice are an improved tool for modeling of human *S*. *aureus* infection.

## Introduction

*Staphylococcus aureus* is an aggressive human pathogen that causes a wide range of diseases and represents a major threat to public health. *S*. *aureus* is the most common cause of bacterial skin and soft tissue infection in the United States and is responsible for over 70% of soft tissue infections treated in emergency rooms [1]. Staphylococcal soft tissue diseases range from superficial infections such as impetigo and abscesses to complicated and life threatening infections such as myositis, pyomyositis, and necrotizing fasciitis.

Numerous animal models have been developed to study *S*. *aureus*-host interaction and to interrogate potential therapeutics against *S*. *aureus* infections. Though these animal models have advanced our understanding of the interaction between *S*. *aureus* and the host, the models have garnered increased scrutiny as translational tools because they have not adequately addressed important issues related to human *S*. *aureus* infections. For example, an expanding list of *S*. *aureus* factors, including LukAB, HlgAB, HlgCB, and Panton-Valentine leukocidin (PVL), show selective affinity for human but not murine receptors [2,3]. Furthermore, all active or passive immunizations developed in mice and taken into human clinical trials have failed to show significant benefit to date [4]. As a consequence, there has been growing consensus that the mouse model does not closely mimic human staphylococcal diseases, and may not represent the best tool to study human *S*. *aureus* pathogenesis or therapeutics [5].

A fundamental problem of the mouse model is that, compared to human infection, a significantly higher inoculum of *S*. *aureus* is required to reproducibly establish pathology in various organs. For skin infection, approximately 10^7^ CFU are needed to induce dermonecrosis in the absence of a foreign object [6]. Though the minimum dose needed to induce skin lesion in human subjects is not known, limited data suggest that human *S*. *aureus* skin infection could be established with as few as 10^4^ CFU *S*. *aureus*, and reproducibly with 10^5^ to 10^6^ CFU [7,8]. The use of higher inocula to induce mouse infection could have unintended consequences on the interpretation of *S*. *aureus* pathophysiology.

For the study of host-pathogen interaction, the mouse model represents an imperfect tool to investigate human tropic *S*. *aureus* factors. As a prime example, PVL, a two-component toxin secreted by most strains of community-associated methicillin-resistant *S*. *aureus* (CA-MRSA), has tropism for human polymorphonuclear leukocytes (PMN), but has an unresolved virulence role based on animal studies [9]. Epidemiological studies link PVL to more severe clinical cases of pneumonia, furunculosis, and abscesses [10], but PVL virulence has been shown to be limited in most murine studies unless an extremely high inoculum is used (10^9^ CFU) [11–14]. Investigations of PVL virulence function in other animal models have also been conflicting. In particular, PVL exhibits pathogenic functions in some but not other rabbit infection studies [9]. Recent identification of C5aR as the primary receptor for PVL has provided clarification on the animal data, as PVL binds with high affinity to human C5aR, to a lesser extent to rabbit C5aR, and minimally to murine C5aR[15]. However, it is still unclear whether PVL has pathogenic functions in human *S*. *aureus* infection.

The problems associated with murine models of staphylococcal infection are not unique, as many important pathogens such as HIV, hepatitis B and C, and *Salmonella enterica* serovar Typhi display unique human tropism [16,17]. To address these limitations, mice with human immune system—so called "humanized" mice—have emerged, and hold great promise as powerful tools for translational research [18]. These mice accept human hematopoietic cells (CD34^+^Lin^-^) and give rise to human innate and adaptive immune cells, yielding mice with a “humanized” immune system. Of the various types of humanized mouse models, non-obese diabetic (NOD)/severe combined immune deficiency (SCID)/IL2rγ^null^ (NSG) mice have now emerged as one of the favored models for the study of host-pathogen interactions [18]. The model has been successfully adopted for the study of certain infections such as HIV, *S*. *typhi*, and mycobacteria. Reconstituted human cells in these mice respond to infections by markedly elevating human pro- and anti-inflammatory cytokines [17,19]. Recently, the model has been adopted to study human T cell activation and cell death in response to *S*. *aureus* septicemia [20]. Based on these studies, we investigated whether humanized NSG mice more closely model human *S*. *aureus* infection.

## Results

### Humanized NSG mice show enhanced susceptibility to *S*. *aureus* skin and soft tissue infection

**Fig 1:**
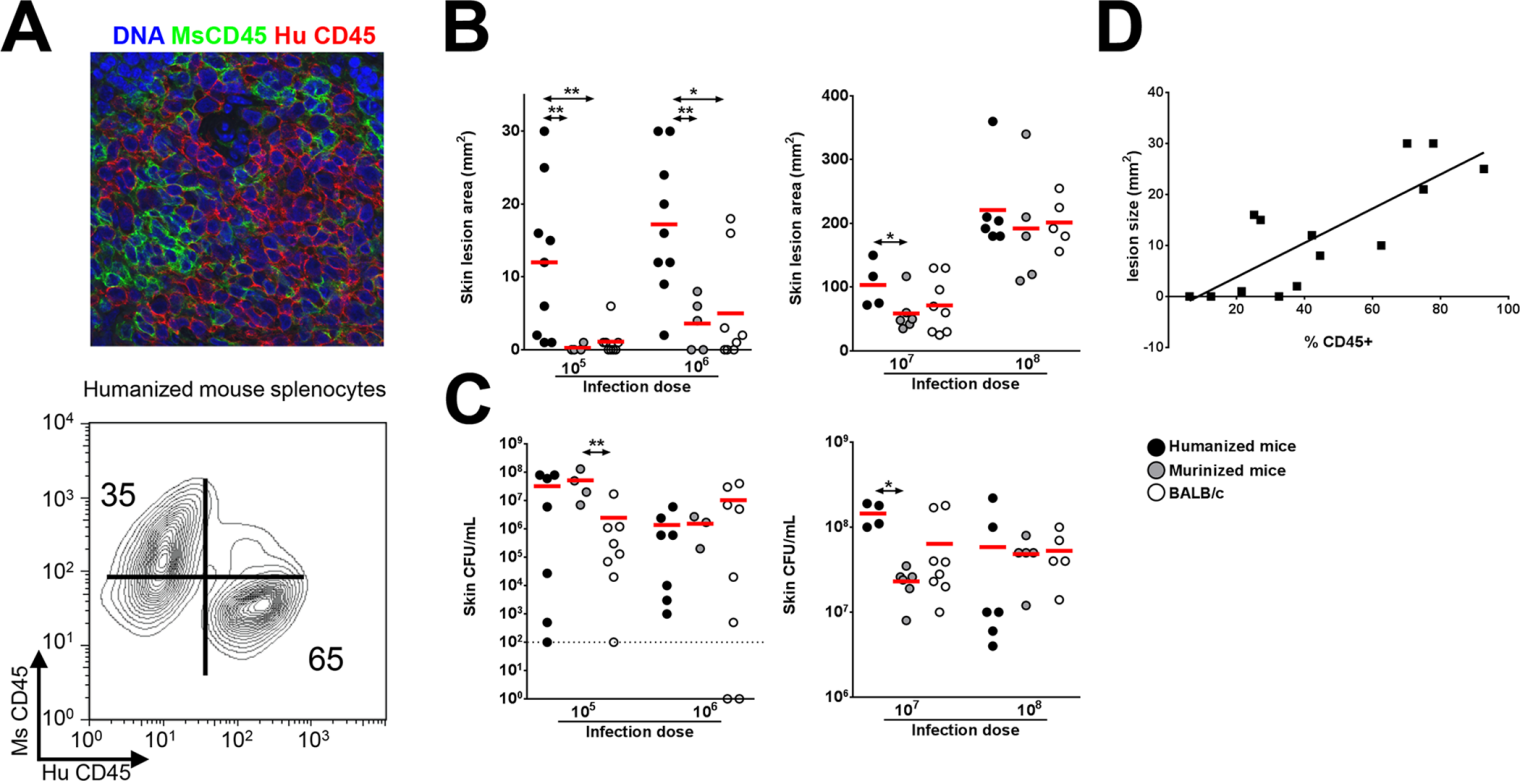
Humanized NSG mice show enhanced susceptibility to *S*. *aureus*-induced skin lesions. (A) Fluorescence confocal imaging of humanized mouse spleen showing separate mouse CD45 and human CD45-expressing cells (top) and flow cytometry contour plot showing % engraftment in humanized mice (bottom). (B-C) Humanized mice and control mice were infected s.c. with 10^5^ to 10^8^ CFU of *S*. *aureus*. On d 3 post-infection, (B) skin lesion size and (C) bacterial burden were analyzed. (D) Mice were infected with 10^6^ CFU of *S*. *aureus* and lesion sizes on d 3 post-infection were plotted against % engraftment. Shown is the best fit linear regression line (R^2^ = 0.65). Red bar = mean, *: *p* <0.05, **: *p* < 0.01.

Humanized NSG mice were generated using an established protocol [21], and engraftment was quantified by staining splenic cells with a monoclonal antibody specific for human nuclei and verified by immunostaining with anti-human and anti-mouse CD45 antibodies (Fig 1A and S1 Fig). Engraftment rates were 70.59 ± 4.02%, which are consistent with results reported by another group [21]. The percentages of human cell subsets in the spleen are shown in S1 Table and demonstrate a predominance of T and B cell subsets over myeloid subsets, consistent with published data [21], though in our study the percentage of CD3^+^ T cells was higher than the percentage of CD20^+^ B cells, unlike the previous study. For infection experiments, control mice consisted of either NSG mice engrafted with murine bone marrow cells (designated as murinized mice) or wild type (WT) BALB/c mice which are congenic with NSG mice. Mice with greater than 40% human CD45^+^ cell engraftment were used in all experiments except for the experiment shown in Fig 1D where engraftment efficiency is correlated to lesion size. To determine the susceptibility of the humanized NSG mice to *S*. *aureus* infection, we infected mice subcutaneously with inocula ranging from 1 x 10^5^ to 1 x 10^8^ CFU. Based on work from our lab and other groups, peak lesion size is documented on d 3 after infection [6,12]. We therefore sacrificed the animals on d 3 for various analyses. Using inocula of 1 x 10^5^ and 1 x 10^6^ CFU, all humanized mice exhibited visible skin lesions (Fig 1B). By comparison, murinized NSG or BALB/c mice exhibited minimal skin lesions at an inoculum of 1 x 10^5^ CFU, while approximately 50% of infected control mice showed visible skin lesions at 1 x 10^6^ CFU. Upon increasing the inocula to 1 x 10^7^ and 1 x 10^8^ CFU, all murinized mice and BALB/c mice showed dermonecrosis (Fig 1B). In spite of the large difference in susceptibility to lesion formation, humanized and control mice generally did not exhibit significant differences in bacterial burden across the range of inocula (Fig 1C). Because many pups injected with hCD34^+^ cells died from maternal neglect or cannibalism in our colony, some of the murinized or humanized NSG groups were small, and for those groups, the data need to be interpreted cautiously.

It was notable that there was significant variation in lesion sizes in humanized mice infected with lower doses of *S*. *aureus* (Fig 1B). To determine whether the variation was related to the rate of engraftment, mice with a broad distribution of human CD45^+^ cell engraftment were infected with *S*. *aureus* and the engraftment efficacy was correlated to lesion size. As shown in Fig 1D, there was good correlation (R^2^ = 0.65) between lesion size and engraftment efficacy.

To examine whether increased susceptibility of humanized mice to gross skin pathology was associated with increased human pro-inflammatory cytokine release, several cytokines were measured from the homogenized skin tissue. Elevated levels of human IL-8, IL-17A, TNFα, and IL-6 were detected (S2 Fig), consistent with prior reports that humanized NSG mice secrete specific human cytokines in response to bacterial pathogens [17,19]. The contribution of individual human cytokines and chemokines to skin pathology will be an area of interest in future studies.

### PVL induces dermopathology in the humanized NSG mouse model

**Fig 2:**
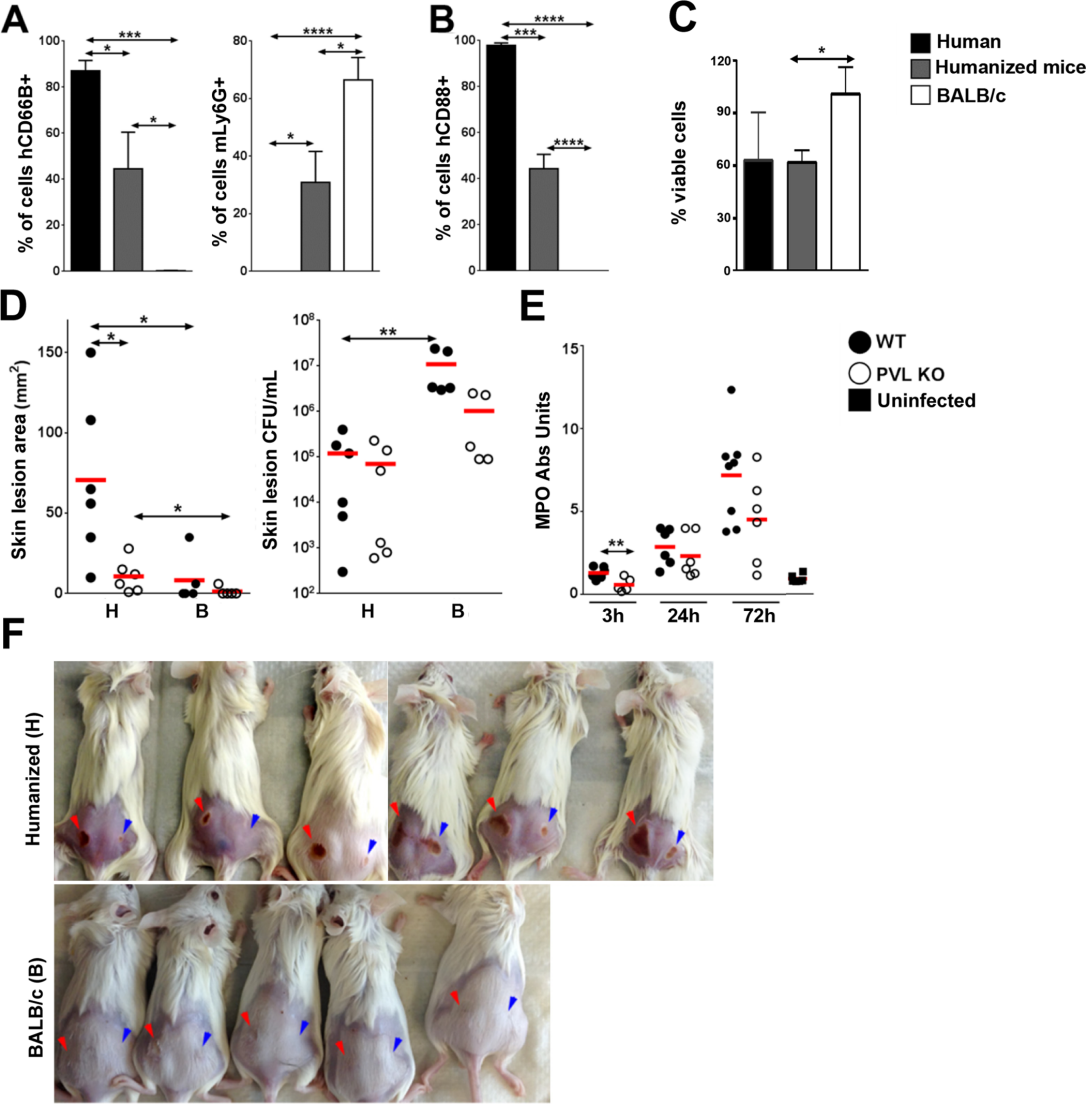
Humanized NSG mice are more susceptible to PVL-induced dermonecrosis. PMN were isolated from the blood of human volunteers and bone marrow of humanized mice or BALB/c mice. (A) Percentage of human CD66B^+^ and murine Ly6G ^+^ cells in the PMN preparations (n = 4). (B) Percentage of hC5aR^+^ cells in the PMN preparations (n = 4). (C) The PMN preparations were incubated with 100 ng/mL rPVL. After 3h, the percentages of viable cells were calculated based on MTT values using untreated PMN isolated from the respective hosts as standards (n = 4). (D—F) Humanized and control mice (n = 5–6 per group) were infected on the left flank with 10^6^ CFU WT *S*. *aureus* and on the right flank with 10^6^ CFU PVL^-^ isogenic mutant strain. The mice were sacrificed on d 3 post-infection. Shown are (D) skin lesion size and bacterial burden (H: humanized mice, B: BALB/c mice) and (E) MPO activity in infected humanized mice. (F) Visual representation of skin lesions induced by WT *S*. *aureus* (red arrow head) or the PVL^-^ mutant (blue arrow head), *: *p* <0.05, **: *p* < 0.01, ***: *p* < 0.005, ****: *p* < 0.001.

Published studies have established that human PMN are exquisitely sensitive to PVL-induced cytolysis, whereas murine PMN are relatively unresponsive to the toxin as determined by chemokine secretion and cytolysis [15,22]. This difference has been attributed to poor binding of the LukS-PV component of PVL to murine C5aR compared to binding to human C5aR [15]. We investigated the interaction of PVL with PMN isolated from humanized NSG mice, human volunteers, and BALB/c mice. First, PMN from the various sources were analyzed by flow cytometry for expression of the human neutrophil marker, hCD66B, and the murine granulocyte marker, Ly6G. As shown in Fig 2A, PMN prepared from the bone marrow of humanized mice yielded 45% human PMN based on hCD66B staining and 31% murine PMN based on Ly6G staining, which is consistent with published reports that NSG mice have a modest but significant number of murine PMN [23]. By contrast, PMN prepared from human volunteers were 87% positive for hCD66B and did not stain for Ly6G. PMN from the bone marrow of BALB/c mice yielded 67% Ly6G-positive cells and had an insignificant percentage of hCD66B-positive cells. Consistent with these findings, PMN obtained from the humanized mice were 44% positive for the PVL receptor hC5aR, compared to 0% positive for PMN from BALB/c mice (Fig 2B). When the cellular preps from the various sources were exposed to recombinant PVL (rPVL), murine PMN showed little loss of viability (Fig 2C) and minimal CXCL1 (IL-8 or KC) response (S3 Fig). In comparison, PMN preparations from humans and humanized mice exhibited significant sensitivity to rPVL as measured by cell viability (Fig 2C) and IL-8 production (S3 Fig). These data show that human PMN generated in humanized NSG mice, like human PMN, are responsive to PVL.

The importance of PVL in human infection remains controversial. Using inocula (10^7^ to 10^9^ CFU) of paired isogenic PVL+/- *S*. *aureus* strains, including the isogenic pair used in this study, we and others have been unable to demonstrate dermonecrosis attributable to PVL in various strains of mice [11–13]. To examine whether PVL has a pathogenic role in the humanized NSG mice, we infected mice with both strains of *S*. *aureus*: the left flank was injected with a WT PVL^+^ *S*. *aureus* strain and the right flank with the isogenic PVL^-^ mutant strain. An inoculum of 10^6^ CFU was chosen to allow a clear difference in lesion size to be visualized. As shown in Fig 2D and 2F, WT *S*. *aureus* induced larger skin lesions compared to PVL^-^ mutant bacteria in the humanized mice (70.7 ± 20.7 mm^2^ vs 10.7 ± 4.1 mm^2^), but CFU burdens were not different between the WT and PVL^-^ mutant groups (Fig 2D), indicating that induction of dermonecrosis was not secondary to changes in bacterial burden induced by PVL. Apart from the dermonecrosis, underlying tissues evaluated by histology did not show differences in inflammatory scores between WT and PVL^-^ groups (mean 2.0 vs 2.0). Myeloperoxidase (MPO) activity, an approximate measure of PMN infiltration [24], was transiently higher in the PVL group at 3 h post infection, but was not significant at any other time points (Fig 2E). Measurement of several human cytokines and chemokines associated with mouse skin infection failed to show a significant difference between the WT and PVL^-^ mutant groups (S4 Fig).

### PVL induces dermonecrosis in NSG mice adoptively transferred with human neutrophils

**Fig 3:**
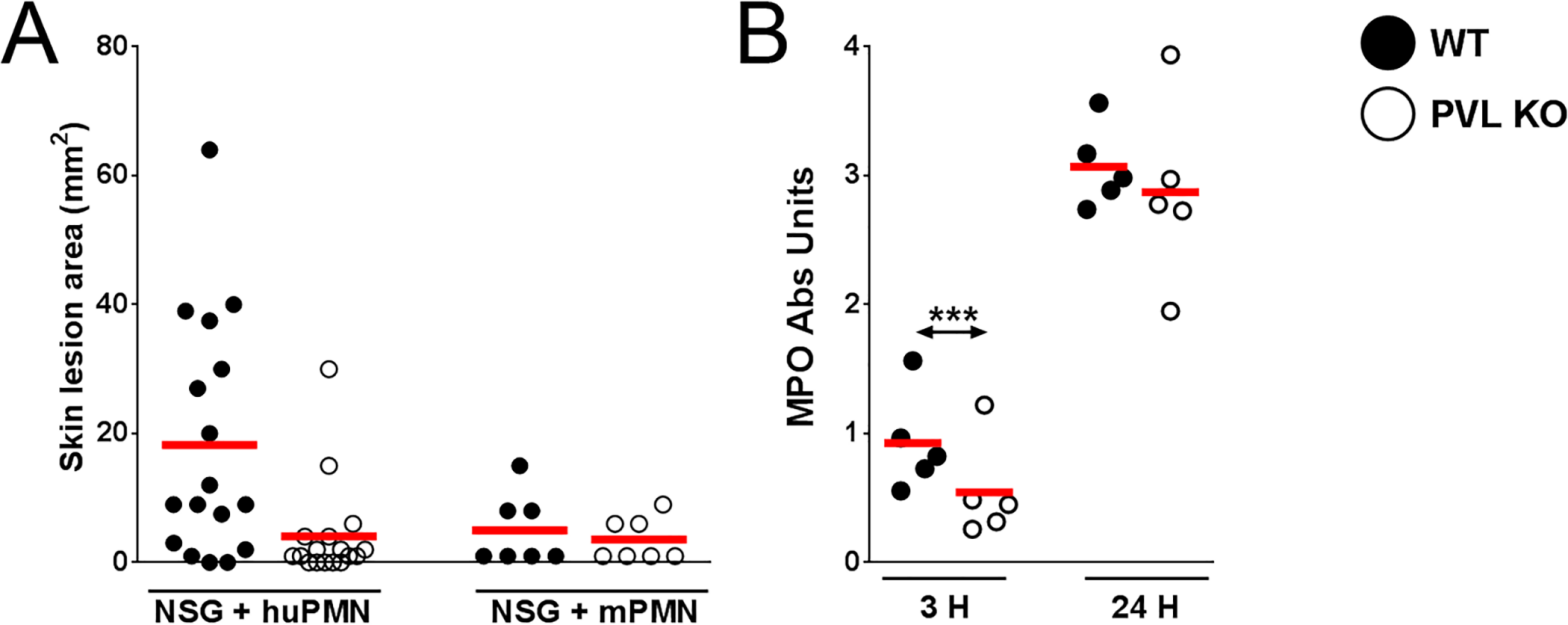
PVL contributes to dermonecrosis in NSG mice adoptively transferred with human PMN. NSG mice were injected i.v. with 5 x 10^6^ human PMN (n = 17) or mouse PMN (n = 7). Three hours later, the mice were infected on the left flank with 10^6^ CFU WT *S*. *aureus* and on the right flank with 10^6^ CFU PVL^-^ isogenic *S*. *aureus*. The mice were sacrificed on d 3 post-infection. Shown are (A) skin lesion sizes on d 3 and (B) MPO activity at 3 h and 24 h. Red bar = mean, ***: *p* < 0.005.

It has been hypothesized that human PMN play a central role in PVL-mediated injury. Among immune cells, human PMN express the highest level of C5aR on their surface [15] and are the most susceptible to the cytotoxic effect of PVL. We sought to address the role of PMN in PVL-related pathology by performing cellular depletion experiments, but were unable to identify a source of depleting antibodies against human PMN. As an alternative, we injected NSG mice i.v. with PMN isolated from human volunteers or BALB/c mice. After 3 h, we infected the mice with 10^6^ CFU isogenic WT and PVL^-^ *S*. *aureus*. Overall, mice injected with human PMN and WT bacteria produced prominent lesions in only about half of the animals (Fig 3A). Therefore, this simplified model of humanized mice is not as robust as NSG mice injected neonatally with human CD34^+^ cells. However, consistent with findings from the humanized NSG mice, PVL^+^ *S*. *aureus* induced significantly larger lesions compared to the isogenic PVL^-^ mutant (Fig 3A), while PVL^+^ and PVL^-^ *S*. *aureus* induced smaller lesions of comparable sizes in NSG mice injected with mouse PMN. As was observed in infected humanized NSG mice, there was a transient increase in MPO at 3 h but not at 24 h post-infection in NSG mice adoptively transferred with human PMN (Fig 3B). Evaluation at the infection site for CFU and human cytokines secreted by human PMN also showed no differences between the WT and PVL^-^ mutant groups (S5 Fig).

Overall these data are compatible with the interpretation that PVL contributes to the formation of dermonecrotic lesions without conferring a survival advantage to *S*. *aureus*. Consistent with the pathologic findings in humanized NSG mice, PVL^+^ *S*. *aureus* induced significant larger skin lesions in mice engrafted with human PMN alone when compared to the PVL^-^ isogenic strain. Our results indicate that human PMN and their interaction with PVL are potentially important for MRSA immunopathology.

### Effect of human C5aR neutralization on PVL cytotoxicity i*n vitro* and *S*. *aureus* skin infection

**Fig 4:**
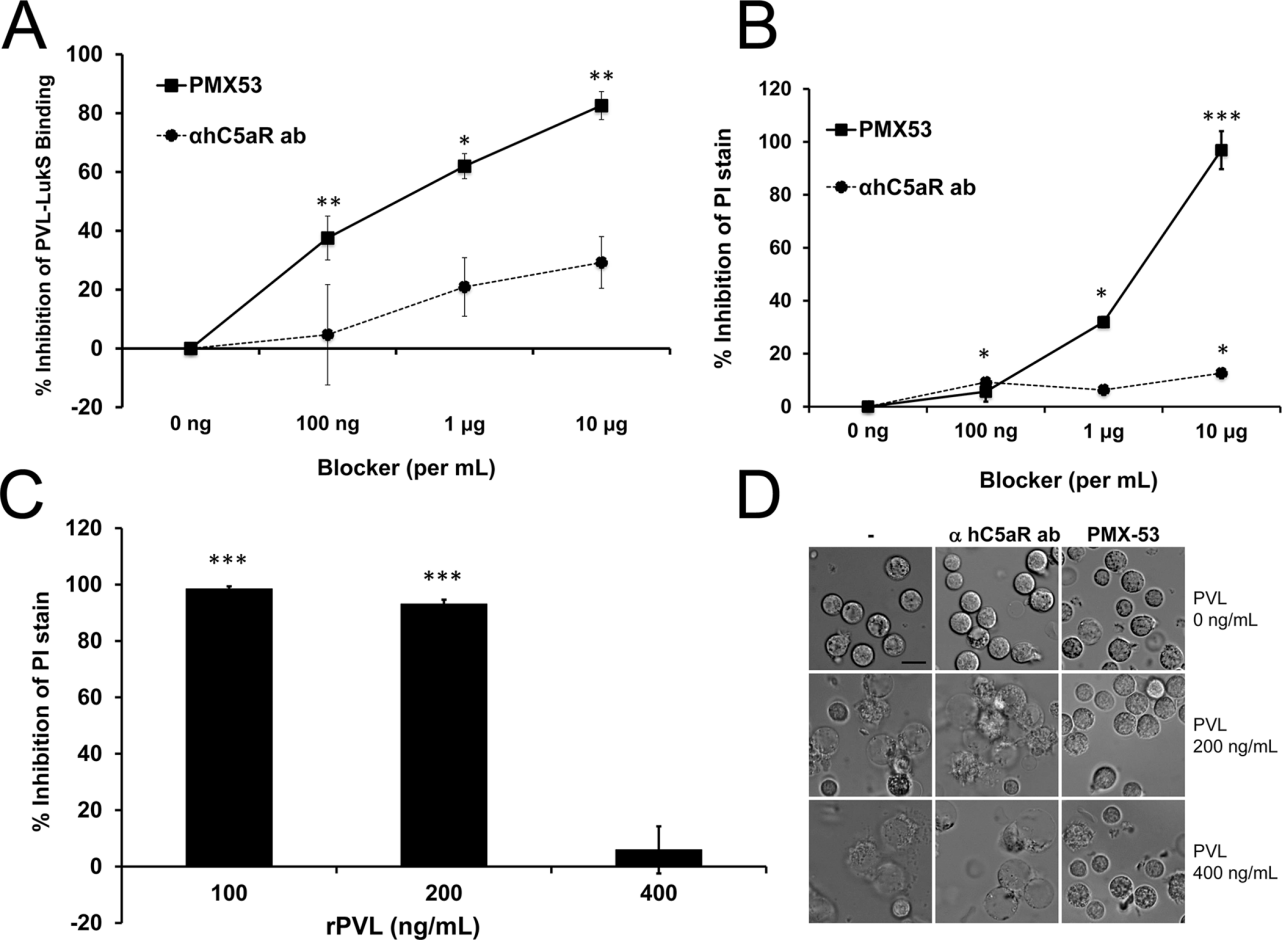
PMX53 inhibits PVL-mediated pore formation and cytotoxicity *in vitro*. Human PMN were incubated with C5aR blockers (PMX53 or hC5aR antibody) and then exposed to rLukS-PV with or without rLukF-PV. (A) PVL binding to PMN was measured and % binding inhibition vs untreated cells was calculated. rLukS-PV:100 ng/mL. (B-C) PVL-induced pore formation was assayed by PI staining. rPVL:200 ng/mL in B. (D) PVL-induced PMN cytotoxicity in the presence of 10 μg/mL blockers. Representative images are shown. Scale: 10 μm. (n > 3 for A-D). *: *p* <0.05, **: *p* <0.01; ***: *p* < 0.005.

Because hC5aR serves as the primary receptor for PVL, the humanized NSG mouse represents a unique tool to address the question whether blockade of hC5aR could ameliorate PVL^+^CA-MRSA diseases. We first sought out inhibitors of hC5aR that also blocked PVL binding. Based on the study by Spaan and colleagues, two antibodies to hC5aR, both directed at the N-terminal domain of hC5aR, competed with LukS-PV for binding to PMN albeit a high concentration of PVL (313 nM) was required [15]. We tested one of the antibodies (S5/1) for inhibition of PVL activities, including binding to PMN, pore formation, and cytotoxicity, but found the antibody to be a relatively ineffective blocker (Fig 4A, 4B and 4D). Alternatively, we tested a well-characterized synthetic peptide blocker of hC5aR, PMX53, which has demonstrated C5aR inhibitory activity *in vitro* and in various models of C5a–related diseases [25,26]. PMX53 binds with high affinity to hC5aR and with relatively low affinity to mC5aR [27]. In the human PMN assays, PMX53 showed good inhibition of PVL binding, pore formation, and cellular toxicity compared to the S5/1 antibody to hC5aR (Fig 4A–4D), but the effect was modest when a higher concentration of PVL (400 ng/mL) was used (Fig 4C).

**Fig 5:**
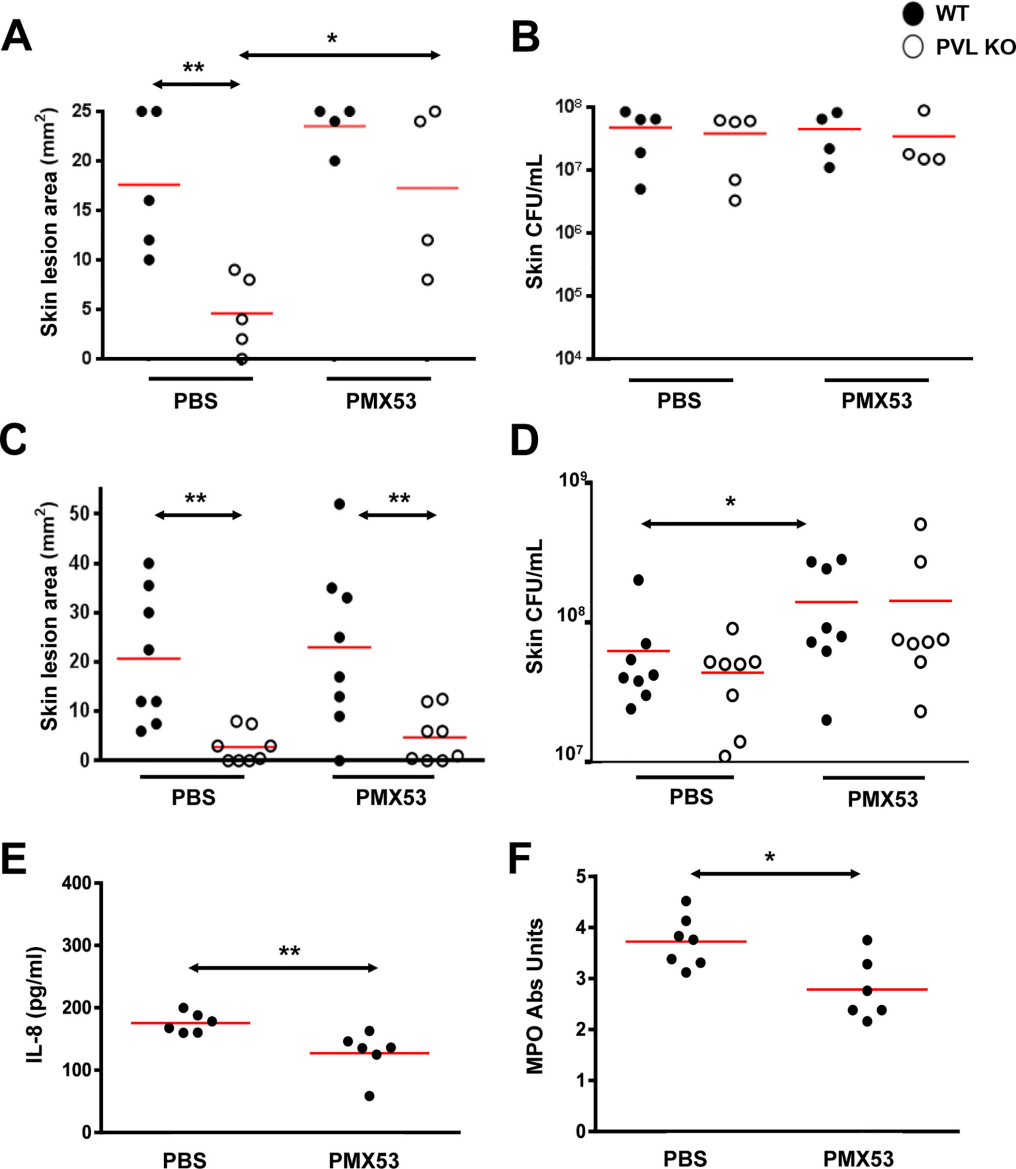
PMX53 reduces the size differences of lesions induced by WT and PVL^-^ *S*. *aureus*. Humanized NSG mice were injected i.p. with PBS or PMX53 (5 mg/kg/d). One hour later, the mice were infected on the left flank with ~2 x 10^6^ WT *S*. *aureus* and on the right flank with ~2 x 10^6^ CFU PVL^-^ isogenic *S*. *aureus*. PBS or PMX53 was subsequently administered once daily. (A) Skin lesions and (B) CFU were measured on d 3. Note 2 mice treated with PMX53 died of an unknown cause prior to d3 and were not included in the analyses. (C—F) Humanized NSG mice were infected with ~2 x 10^6^ WT *S*. *aureus* and treated 3 h later and then daily with PBS or PMX53 (5 mg/kg/d) i.p. (C) Skin lesion sizes and (D) bacterial burden on d 3. (E) IL8 release and (F) MPO at the site of infection after 24 h. Red bar = mean, *: *p* <0.05, **: *p* <0.01.

To address the pathogenic role of human C5aR-PVL interaction *in vivo* and test the efficacy of C5aR blockade as a treatment for *S*. *aureus* skin infection, we next infected humanized NSG mice with WT and isogenic PVL^-^ mutant *S*. *aureus* strains, and treated the mice either 1 h prior or 3 h after infection with PMX53. Based on several published studies, daily systemic injection of PMX53 at 1 mg/kg effectively blocked various disease manifestations attributable to C5a, but has well-documented immunosuppressive effects [25,26]. Because a relatively high concentration of PMX53 was required to block PVL cytolytic activity *in vitro*, we selected a PMX53 dose of 5 mg/kg i.p given once daily. As shown in Fig 5A, administration of PMX53 versus PBS prior to infection did not improve skin lesion severity in mice infected with WT PVL^+^ *S*. *aureus*, but reduced the lesion size difference between the PVL^+^ and PVL^-^ infection groups, suggesting blocking of PVL effect by PMX53. For skin infection induced with the isogenic PVL^-^ *S*. *aureus*, PMX53 treatment increased lesion sizes, indicating a potential detrimental effect of neutralizing C5aR-C5a interaction.

When PMX53 was given 3h after infection, the treatment no longer affected the lesion size difference between the PVL^+^ and PVL^-^ infection groups (Fig 5C). In addition, PMX53 no longer affected lesion sizes in mice infected with either PVL^+^ and PVL^-^ *S*. *aureus*, but instead promoted increased *S*. *aureus* survival in the skin, compared to PBS treatment (Fig 5D). Consistent with the hypothesis that immunosuppression induced by C5aR blockade permitted survival of the bacteria, PMX53 treatment reduced IL8 and recruitment of PMN (Fig 5E and 5F). Injection of mice with the S5/1 antibody also led to increased CFU (S6 Fig) without affecting lesion size (average 20 mm vs 17 mm for PBS vs S5/1 groups), thereby corroborating the adverse effect of C5aR inhibitors on *S*. *aureus* infection. It is noteworthy that in mice infected with PVL^-^ *S*. *aureus*, PMX53 administered prior to infection increased lesion sizes without affecting CFU burden (Fig 5B) whereas PMX53 administered after infection increased bacterial burden but did not affect lesion size. Both effects could be interpreted as outcomes of immunosuppression induced by C5aR blockade. However, it is not clear why different outcomes were observed when the timing of treatment was different.

## Discussion

In recent years, investigation of *S*. *aureus* toxins has uncovered a number of human immune receptors that serve as receptors for the bacterial toxins, for example LukAB-CD11b and LukED-CCR5 [2]. In the report describing LukED virulence functions, CCR5-deficient mice were shown to be resistant to a lethal challenge with LukED^+^ *S*. *aureus*, suggesting that blockade of host immune receptors could be a generalized strategy to block *S*. *aureus* toxin effects to ameliorate *S*. *aureus* infection [2,15]. In our study, PMX53 treatment appears to exacerbate infection as may be expected from blockade of a major innate immune receptor. Because the binding sites for C5a and PVL are overlapping but different, it may still be feasible to find an inhibitor that blocks PVL-hC5aR interaction without interfering with normal C5a immune functions. Of note, a recently published study has identified several additional C5aR blockers that inhibit PVL cytolytic activity *in vitro* [28]. However, our data suggest that administration of PMX53 after infection is not effective at reducing pathology and therefore the utility of this approach may be limited.

It has become increasingly clear that mice are problematic as models to study human *S*. *aureus* infection. As was suggested in a recent review of *S*. *aureus* interaction with PMN, most molecules secreted by *S*. *aureus* to combat the phagocyte show clear human specificity [15]. Therefore, the *in vivo* contribution of specific *S*. *aureus* factors to staphylococcal pathophysiology is difficult to establish using current animal models. To overcome the deficits associated with the mouse models for studies of human tropic factors, some investigators have turned to transgenic mice that express one human molecule. For example, Pishchany and colleagues have performed infection experiments in mice expressing transgenic human hemoglobin to demonstrate the virulence role of the *S*. *aureus* hemoglobin receptor IsdB [29]. However, a possible drawback of using mice expressing one human molecule is that the impact of interaction between other *S*. *aureus* factors and their human receptors would not be measured in the model. A recent study has begun exploring *S*. *aureus* pathogenesis in humanized NSG mice. In that study, the authors demonstrated increased mortality of the humanized mice to *S*. *aureus* compared to WT mice. The study went on to demonstrate that T cells from humanized mice are readily activated and undergo apoptosis with *S*. *aureus* infection [20]. In our study, we have shown that humanized NSG mice exhibit several additional qualities that make them particularly attractive for human translational studies. First, the mice are susceptible to *S*. *aureus*-induced dermonecrosis at an inoculum that is compatible with human infection [7,8]. Additionally, the model facilitated the investigation of human-tropic bacterial factors such as PVL, permitted testing of specific human therapeutics, and could be amenable to adoptive transfer of human cells. Because many of the human receptors for *S*. *aureus* factors are expressed on immune cells, the humanized NSG mice provide a unique tool to study the virulence functions of *S*. *aureus* factors. In the humanized skin infection model, even in the absence of PVL, *S*. *aureus* induces skin lesions that appear larger than lesions in normal mice, suggesting that other human tropic factors likely have a role in pathogenesis. Two particular factors, HlgBC and CHIPS, selectively bind human C5aR [3,30,31]. HlgBC is a toxin that is also cytolytic to human PMN, but differentially interacts with C5aR compared to PVL [28]. It is unclear based on our data to what extent it contributes to skin pathology in humanized NSG mice. The humanized NSG mouse model would be well suited to address that and similar virulence questions. Going forward, our study and the study by Knop and colleagues [20] have provided a framework to begin exploring unique aspects of *S*. *aureus* vaccine development using these mice as tools. For example, humanized mice could serve as vessels to evaluate the protective effect of serum or blood components isolated from non-vaccinated or vaccinated individuals.

The mechanistic basis for PVL-induced pathology *in vivo* remains to be fully clarified. Infections with PVL^+^ and PVL^-^ *S*. *aureus* did not induce differences in CFU, or pro-inflammatory cytokines or chemokines. PMN recruitment as measured by MPO was significantly different during PVL^+^ and PVL^-^ *S*. *aureus* infections at an early time point, but not different after 24 h, and the significance of that is not clear. Preliminary studies of caspase 3 staining of histology slides did not show obvious differences between infections induced by WT and PVL^-^ mutant *S*. *aureus*. There are additional cytokines uniquely important to human infections that we have not studied. Further investigation into other mechanisms, including human-specific cytokine pathways, is needed to clarify how PVL induces dermonecrosis.

Our study is consistent with prior reports demonstrating that myeloid components of humanized NSG mice closely mimic human innate immune cell functions [23,32]. The myeloid cells developed in humanized mice have been reported to phagocytose and to produce cytokines, and show human innate cell morphology, STAT3 activation with LPS, and expected tissue distribution [23]. In spite of the select infectious models validating the value of the mice in studies of bacterial pathogenesis, the generation of the humanized mouse is costly, variability could result from use of CD34^+^ cells from different donors, and the mouse is immuno-compromised and is unsuitable for certain types of studies. There also remain known and unknown incompatibility issues between human and mouse, which could impact human immune cell development and functions. For example mouse LFA-1 does not bind human ICAM-1 [33], and the major human chemokine IL-8 is not expressed in mice. Both findings have the potential to affect leucocyte recruitment. In addition, many tools available in mice, e.g. knockouts and neutralizing antibodies, are not available in humanized mouse models. With these issues in mind, the value of the model will be significantly enhanced if the model is further validated for its ability to predict human disease pathogenesis.

## Materials and Methods

### Bacterial strains and growth conditions

A CA-MRSA strain (CST5) and its isogenic PVL knockout strain were used for this study [12]. The bacterial strain was routinely cultured on sheep blood agar plates, and colonies with comparable hemolytic phenotype were used for each experiment. Bacteria were grown in tryptic soy broth at 37°C with shaking at 250 rpm.

### Generation of humanized and murinized mice

Eight-week-old immunodeficient NOD.Cg-*Prkdc* ^scid^ Il2*rg* ^tm1Wjl^/SzJ (NOD-*scid* IL2Rg^null^ [NSG]) mice were purchased from the Jackson Laboratory and housed in specific pathogen-free vivarium and maintained on SCIDS breeder diet (Bio-Serv) until 7–10 d prior to infection. Humanized NSG mice were generated using a previously described protocol [21]. Briefly, human umbilical cord blood was collected and CD34^+^ cells were purified using a CD34 microbead kit (Miltenyi Biotechnology) and an AutoMACS system. Samples were analyzed by flow cytometry and CD34^+^ cells of ≥ 90% purity with ≤ 0.1% contaminating CD3^+^ T cells were routinely obtained, as previously described [34]. One-to-three-day old pups were administered 1 x 10^5^ CD34^+^ cells by intrahepatic injection, without prior irradiation. For murinized NSG mice, one- to three-day-old pups were administered 1 x 10^5^ BALB/c bone marrow cells by intrahepatic injection. The pups were returned to their mothers and weaned after 18–21 d. At 16 weeks, blood PMN counts were similar between murinized NSG mice and BALB/c mice (5.8 ± 0.7% versus 5.5 ± 2.1%).

### Adoptive transfer of human or murine PMN into NSG mice

PMN were isolated from healthy human volunteers using Polymorphprep (Axis-Shield) or from mice using Histopaque cell separation media (Sigma) following the manufacturer’s instructions. After hypotonic lysis of red blood cells, the PMN were washed extensively and resuspended in DPBS (Mediatech). PMN (5 x 10^6^) were injected i.v. via the tail vein into 14- to 16- week-old gender- and age- matched NSG mice 3 h prior to infection with *S*. *aureus*.

### Mouse skin infection model

Gender- and age- matched 12–16 week-old humanized, murinized, and BALB/c mice were used for infection experiments. Age- and gender- matched BALB/c mice were purchased from Charles River Laboratories. Overnight bacterial culture was diluted 1:200 in pre-warmed media and incubated at 37°C with shaking at 250 rpm until an A_540_ ~2.5. Bacteria were harvested by centrifugation at 4000 rpm for 10 min at 4°C, and then washed twice with an equal volume of DPBS. Bacteria were then suspended in DPBS at a concentration of ~10^6^–10^9^ CFU/mL, and 100 μL of the suspension was subcutaneously injected into shaved flank. Injections were performed with careful visualization of the needle to assure that the injections were not intramuscular. All animal experiments were approved by the Cedars-Sinai Committee on the Use and Care of Animals and performed according to accepted veterinary standards.

### *In vivo* blockade of hC5aR

Twelve to 16 week old humanized NSG mice were infected as described above and then injected i.p. with PBS, 5 mg/kg PMX53 (AcF-[OP (D-Cha) WR] (AcetylPhe; Orn-Pro-D-cyclohexylalanine-Trp-Arg), > 95% purity, GL Biochem), or 8 mg/kg mouse anti-hC5aR (Abd Serotec) or isotype control (Abd Serotec) in 500 μL volume. The C5aR blockers or controls were administered 1 h prior or 3 h post infection, and then at 24 h and 48 h post infection.

### Determination of skin lesion size, tissue bacterial burden, MPO activity, and cytokine and chemokine levels

Following euthanization, skin lesions were measured as previously described [12]. The skin lesions were excised and homogenized in 1 mL of DPBS-Triton X-100 (0.05%) plus protease inhibitor cocktails (Roche). CFU determination was performed as previously described [12]. The homogenized suspension was centrifuged at 15,000 x *g* for 10 min, and supernatants were collected and stored at -80°C for subsequent analysis by MPO assay and ELISA. Human IL-8 (R & D Systems), IL-17A, TNFα, IL-1β and IL-6 (BioLegend) ELISAs were performed according to the manufacturers’ instructions. MPO activity was determined using a modification of an established protocol [35]. Briefly, homogenized skin lesion supernatants were diluted in DPBS and added to wells in 96 well plates. Fifty microliters of 3,3',5,5'-tetramethylbenzidine (TMB) (Thermo Scientific) were added to each well. After incubation at room temperature for 30 min, 50 μL of 2N H_2_SO_4_ were added to each well and absorbance at 450 nm was determined using a microplate reader. The results were expressed as arbitrary units (sample reading at A_450_—blank reading at A_450_).

### *In vitro* PMN functional assays

#### PMN isolation

Human PMN were isolated from healthy human volunteers using Polymorphprep according to the manufacturer’s instructions. The isolated cells were washed with DPBS 3 times and resuspended in 5% FBS-RPMI at 1 x 10^6^ cells/mL for the *in vitro* experiments. PMN from BALB/c and humanized mice were isolated from the bone marrow of individual mice using a previously established method. Briefly, red blood cells were lysed using ddH_2_O, and the marrow cells were washed three times with DPBS. The cells were suspended in 4 mL of DPBS and overlayed carefully on top of 3 mL of lympholyte cell separation media (Cedarlane Laboratories). The cells were centrifuged at 830 x *g* for 25 min without break at room temperature. The cell pellets were collected, washed with DPBS three times, and resuspended in 5% FBS RPMI at 1–2 x 10^6^ cells/mL for *in vitro* experiments.

#### PVL induction of IL8 and KC from neutrophils

One hundred microliters of the resuspended PMN was added to each well in a 96 well plate, and 10 μL of diluted rPVL [12] was added to each well to achieve final concentrations ranging from 0.2 to 100 ng/mL. After 3 h incubation at 37°C in 5% CO_2_, the PMN were centrifuged at 700 x *g* for 5 min and the culture supernatants were transferred to a clean 96 well plate and stored at -20°C for human IL8 and murine KC determination by ELISA (R & D Systems).

#### PVL induced PMN cytotoxicity as measured by MTT assay

PVL-treated PMN were washed with DPBS once and resuspended in 100 μL of 0.5 mg/mL of thiazolyl blue in RPMI. After incubation at 37°C for 30 min, the cells were centrifuged at 700 x *g* for 5 min and the supernatants were carefully removed. The cells were lysed with 100 μL of DMSO and the results were read at 570 nm and subtracted from readings at 650 nm. For standards, a titration of the untreated corresponding cells (human, mouse, and humanized mouse cells) was used.

#### Inhibition of PVL pathologic activities using C5aR blockers

Inhibition of PVL binding to PMN: Human PMN were incubated for 10 min at room temperature with PMX53, anti-hC5aR antibody, or an isotype control, followed by rLukS-PV (100 ng/ml) for 30 minutes at 37°C. PVL binding to PMN was detected using polyclonal antibody to LukS-PV and a FITC-labeled secondary antibody, followed by analysis with flow cytometry.

Inhibition of PVL- mediated pore formation: Human PMN were incubated with PMX53 or anti-hC5aR antibody as described above, then PVL was added at 100–400 ng/mL concentrations. After 1 h at 37°C, the cells were stained with propidium iodide (eBioscience, San Diego, CA) and pore formation was analyzed by flow cytometry.

Inhibition of PVL-mediated neutrophil cytotoxicity: Human PMN were incubated with PMX53 or anti-hC5aR antibody and then with PVL as described above. The PMN were visualized after 3h using a Zeiss Observer.Z1 microscope. For inhibition of PVL binding or pore formation, the percent inhibition is calculated as [(PVL binding / % PI stain in the absence of blockers)–(PVL binding / % PI stain in the presence of blockers)] divided by [(PVL binding in the absence of blockers)—(binding / % PI stain in the absence of PVL)] x 100.

### Hematoxylin-eosin staining, immunofluorescence, and flow cytometry

Infected tissues were excised and fixed in 10% formalin (Medical Chemical Corporation) overnight. Paraffin embedding and H&E staining were performed by the Department of Pathology at Cedars-Sinai Medical Center. For histologic analyses, an overall histology score ranging from 0 to 3 was determined for each sample according to the following: 0: normal tissue without apparent changes, 1: minimal acute inflammation, necrosis and edema, and presence of chronic changes including chronic inflammation, fibroblast proliferation, granulation tissue and fibrosis, 2: moderate acute inflammation, necrosis and edema, and with minimal chronic changes, and 3: marked acute inflammation often involving deep skeletal muscle, necrosis and edema, and with minimal chronic changes.

For immunofluorescence staining, sections were incubated in protein block (PBS containing 10% FBS and 0.3% Triton X-100 [Sigma-Aldrich]) for 1 h at room temperature. The antibodies were diluted in protein block and incubated overnight at 4°C. The following antibodies were used: anti-human CD45 biotinylated (R&D Systems), rat anti-mouse CD45 (AbD Serotec), mouse anti-human nuclei (Millipore), and rabbit anti-staphylococcal protein A (Spa) (Sigma-Aldrich). After three rinses for 5 min each in PBS, samples were incubated for 1 h at room temperature with appropriate Alexa Fluor-conjugated secondary antibodies. Samples were rinsed 3 times with PBS for 5 min at room temperature. After air-drying in the dark, the samples were mounted with Prolong Gold anti-Fade with DAPI (Life Sciences). Three random fields from each sample were imaged using a Nikon A1R-A1 confocal microscope. For the humanized mice, percentage reconstitution rate was reported as the number of cells stained with human nuclei divided by total number of nuclei x 100.

For flow cytometric analyses of splenocytes and isolated PMN, FITC-conjugated anti-human CD66B, CD33, C5aR, and CD3, PE-conjugated anti-human CD20, and PE-conjugated anti-mouse Ly6G and corresponding isotype control antibodies were used (BioLegend). The isolated PMN from human, humanized mice, and BALB/c mice were fixed in 100% methanol at room temperature for 10 min. Prior to incubation with blocking buffer (DPBS, 20% FBS, 20% human donor AB sera [Gemini], 20% goat normal sera [Sigma-Aldrich], 5% BSA [Sigma-Aldrich], and 5 μg/mL DNase [Sigma-Aldrich], in the final concentration) at room temperature for 1 h cells were washed with DPBS twice. Antibodies were added to each sample at a 1:100 dilution in the blocking buffer and incubated at room temperature for 15 min. The cells were rinsed twice in blocking buffer, resuspended in DPBS, and analyzed using a CyAn flow cytometer (Beckman Coulter).

### Statistical analysis

Two-group analysis used either unpaired two-tailed *t*-test or a non-parametric Mann–Whitney U-test in the case of missing normality. Two-way ANOVA with Bonferroni correction was used for comparisons between more than two independent groups. Correlation analysis between lesion sizes and engraftment was performed using linear regression and evaluation of R^2^. GraphPad Prism was used for all analyses, and *p* values less than 0.05 were considered to be statistically significant.

### Ethics Statement

This study was performed under strict accordance with the recommendations from the Guide for the Care and Use of Laboratory Animals. CSMC is accredited by the Association for Assessment and Accreditation of Laboratory Animal Care International (AAALAC), and is in compliance with NIH guideline of laboratory animal care and use. The protocol was approved by the institutional animal use and care committee of the Cedars-Sinai Medical Center (IACUC protocol #3402). All procedures were performed under isoflurane anesthesia, and all efforts were made to minimize suffering. Experimentations using human blood were approved by the Cedars-Sinai Medical Center Institutional Review Board (Pro00009792 and Pro00004485). Anonymized human cord blood was obtained for the purification of CD34+ cells, and consent was exempted by the IRB. Human blood was also obtained from adult volunteers, and written consent was obtained prior to the blood draw. All consent for human blood was informed.

## Supporting Information

S1 Table Human cell chimerism in the spleen of humanized NSG mice at 12–14 weeks. (DOCX)

S1 Fig Flow cytometry analyses of humanized mouse spleens show engraftment of human leukocytes. (A) Histogram of CD34^+^ cells isolated with CD34 microbeads prior to injection into NSG mice. (B) Flow cytometry contour plot of the CD34^+^ fraction showing expression of human CD34 and human CD45. (C) Fluorescence confocal imaging of humanized mouse spleen showing co-localization of human nuclear antigen and human CD45. (D) Histograms of human and mouse CD45 corresponding to the contour plot shown in Fig 1A.
(TIF)

S2 Fig Human pro-inflammatory cytokines and chemokines at the *S*. *aureus* infection site. Humanized NSG mice (n = 4–8) were infected with 10^5^ CFU of *S*. *aureus*. On d 3 post-infection, infected skin samples were homogenized and assayed for human cytokines and chemokines by ELISA. Shown are (A) IL-8, (B) IL-17A, (C) TNFα, and (D) IL-6. UI: uninfected. Red bar = mean, **: *p* < 0.01, ***: *p* <0.005.
(TIF)

S3 Fig Responses of PMN from human volunteers, BALB/c mice, and humanized mice to PVL. PMN were isolated from the blood of human volunteers and bone marrow of humanized mice or BALB/c mice. The PMN preparations were incubated with rPVL. After 3 h, (A) human IL-8 (n = 3, *p* < 0.05, two-way ANOVA) and (B) mouse KC (n = 3, *p* < 0.01, two-way ANOVA) were measured from the PMN supernatants. Human IL-8 and murine KC levels following PMN incubation with medium or 100 ng/mL LPS are also shown. **: *p* <0.01 compared to human; #: *p* < 0.05 compared to mouse (*n* = 3 experiments).
(TIF)

S4 Fig Chemokine and cytokine responses to WT *S*. *aureus* or isogenic PVL^-^ mutant in humanized NSG mice. Humanized mice were infected on the left flank with 10^6^ CFU WT *S*. *aureus* and on the right flank with 10^6^ CFU PVL^-^ isogenic mutant strain. The mice (n = 4–6 mice per group) were sacrificed at various time points post-infection. Shown are (A) Human IL-8, (B) IL-17A, (C) IL-1β, (D) TNF-α, and (E) IL-6 from the infection sites. UI: uninfected. Red bar = mean.
(TIF)

S5 Fig Bacterial burden, and chemokine and cytokine responses to WT *S*. *aureus* or isogenic PVL^-^ mutant in NSG mice adoptively transferred with human neutrophils. NSG mice were injected i.v. with 5 x 10^6^ human PMN. Three hours later, the mice were infected on the left flank with 10^6^ CFU WT *S*. *aureus* and on the right flank with 10^6^ CFU PVL^-^ isogenic *S*. *aureus*, and sacrificed 3 h or 24 h after infection. Shown are (A) bacterial CFU, (B) human IL-8, (C) IL-1β, and (D) TNFα from the infection sites. Red bar = mean.
(TIF)

S6 Fig Anti-hC5aR promotes survival of *S*. *aureus in vivo*. Humanized NSG mice were infected on the left flank with ~2 x 10^6^ WT *S*. *aureus* and on the right flank with ~2 x 10^6^ CFU PVL^-^ isogenic *S*. *aureus*. After 3 h, the mice were injected i.p. with anti-hC5aR or an isotype control antibody (8 mg/kg/d). Bacterial burden was measured on d 3. Red bar = mean.
(TIF)
