# Supplementary material for: Increased Susceptibility of Humanized NSG Mice to Panton-Valentine Leukocidin and Staphylococcus aureus Skin Infection
Source: PLoS Pathog. 2015 Nov 30;11(11):e1005292. doi: 10.1371/journal.ppat.1005292 (PMC4664407; doi:10.1371/journal.ppat.1005292)
Supplement: S1 Table — (DOCX) [file ppat.1005292.s001.docx]

**Supplementary Table S1.** Human cell chimerism in the spleen of humanized NSG mice at 12-14 weeks. Splenocytes from NSG humanized mice were stained with human T, B, and myeloid specific antibodies. Shown are the percentage cell subsets within the hCD45 population (n=4-5).

___________________________________________________________________________

CD3 (T-cell lineage) 60.6 +/- 16.1

CD20 (B-cell lineage) 17.7 +/- 10.1

CD33 bright (Monocytic lineage) 8.2 +/- 0.8

CD66B (granulocytic lineage) 10.0 +/- 3.2

__________________________________________________________________________
