# Supplementary figures and images for: Increased Susceptibility of Humanized NSG Mice to Panton-Valentine Leukocidin and Staphylococcus aureus Skin Infection
Source: PLoS Pathog. 2015 Nov 30;11(11):e1005292. doi: 10.1371/journal.ppat.1005292 (PMC4664407; doi:10.1371/journal.ppat.1005292)

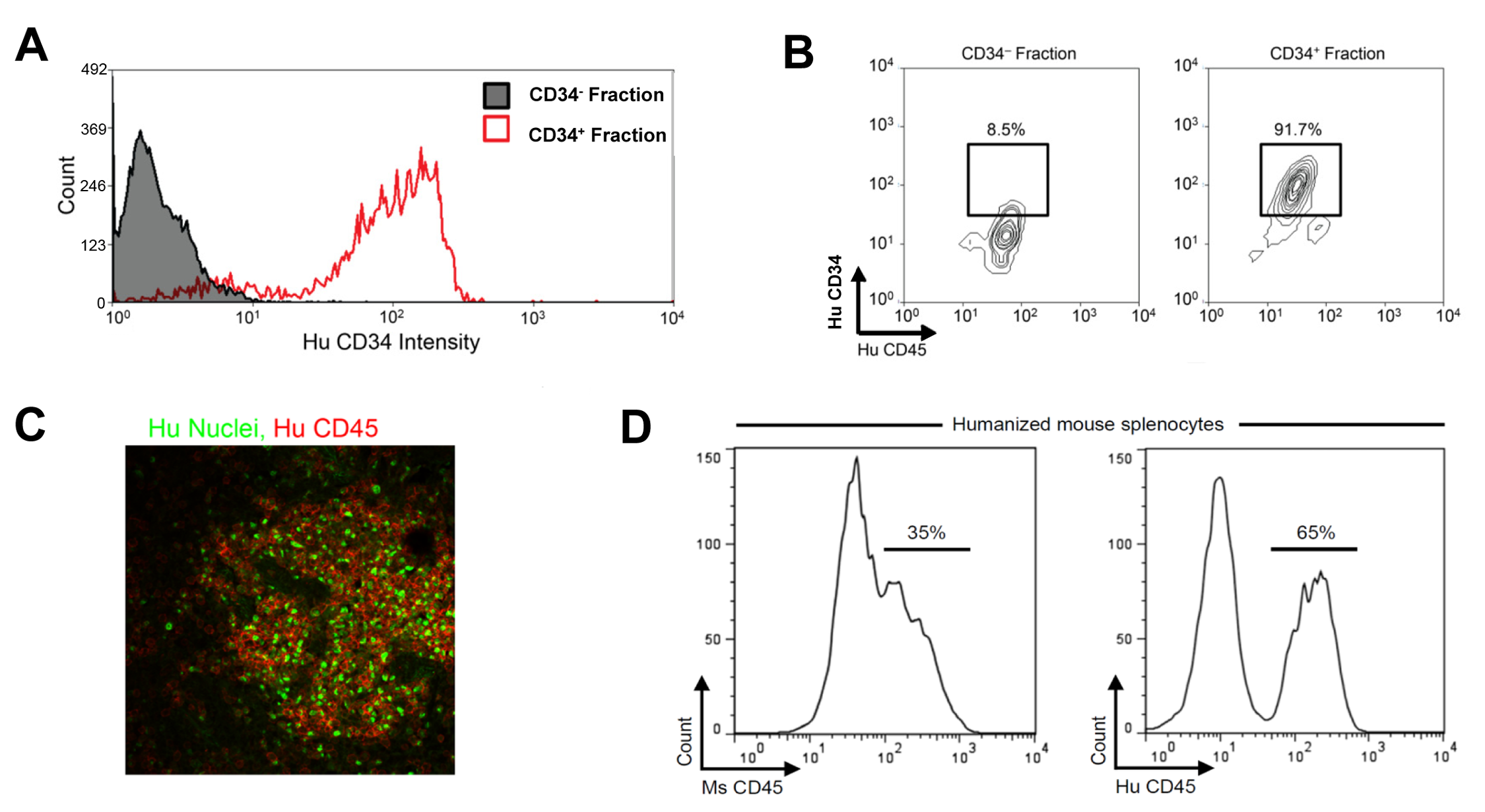

Supplement: S1 Fig — (A) Histogram of CD34+ cells isolated with CD34 microbeads prior to injection into NSG mice. (B) Flow cytometry contour plot of the CD34+ fraction showing expression of human CD34 and human CD45. (C) Fluorescence confocal imaging of humanized mouse spleen showing co-localization of human nuclear antigen and human CD45. (D) Histograms of human and mouse CD45 corresponding to the contour plot shown in Fig 1A. (TIF) [file ppat.1005292.s002.tif]

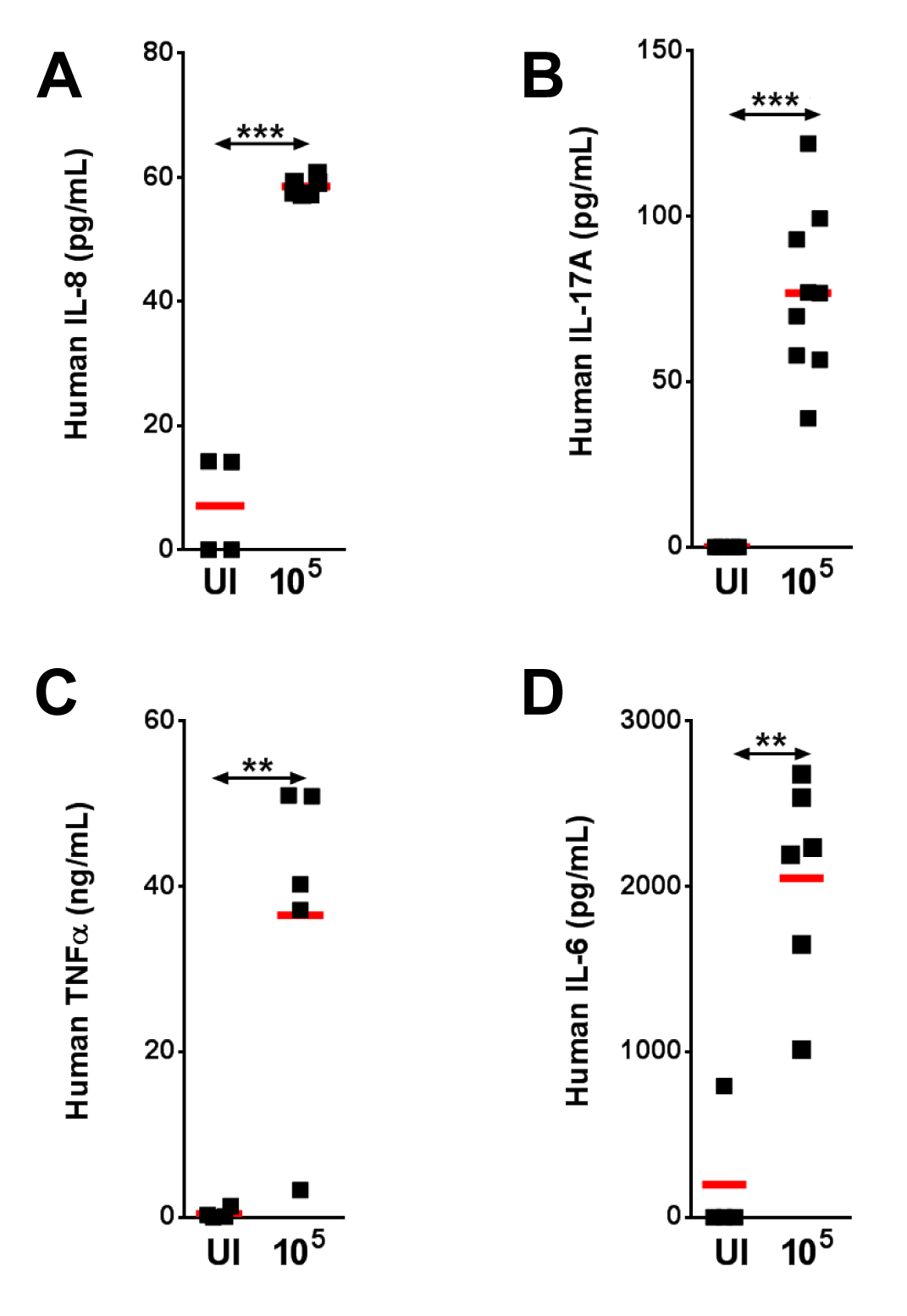

Supplement: S2 Fig — Humanized NSG mice (n = 4–8) were infected with 105 CFU of S. aureus. On d 3 post-infection, infected skin samples were homogenized and assayed for human cytokines and chemokines by ELISA. Shown are (A) IL-8, (B) IL-17A, (C) TNFα, and (D) IL-6. UI: uninfected. Red bar = mean, **: p < 0.01, ***: p <0.005. (TIF) [file ppat.1005292.s003.tif]

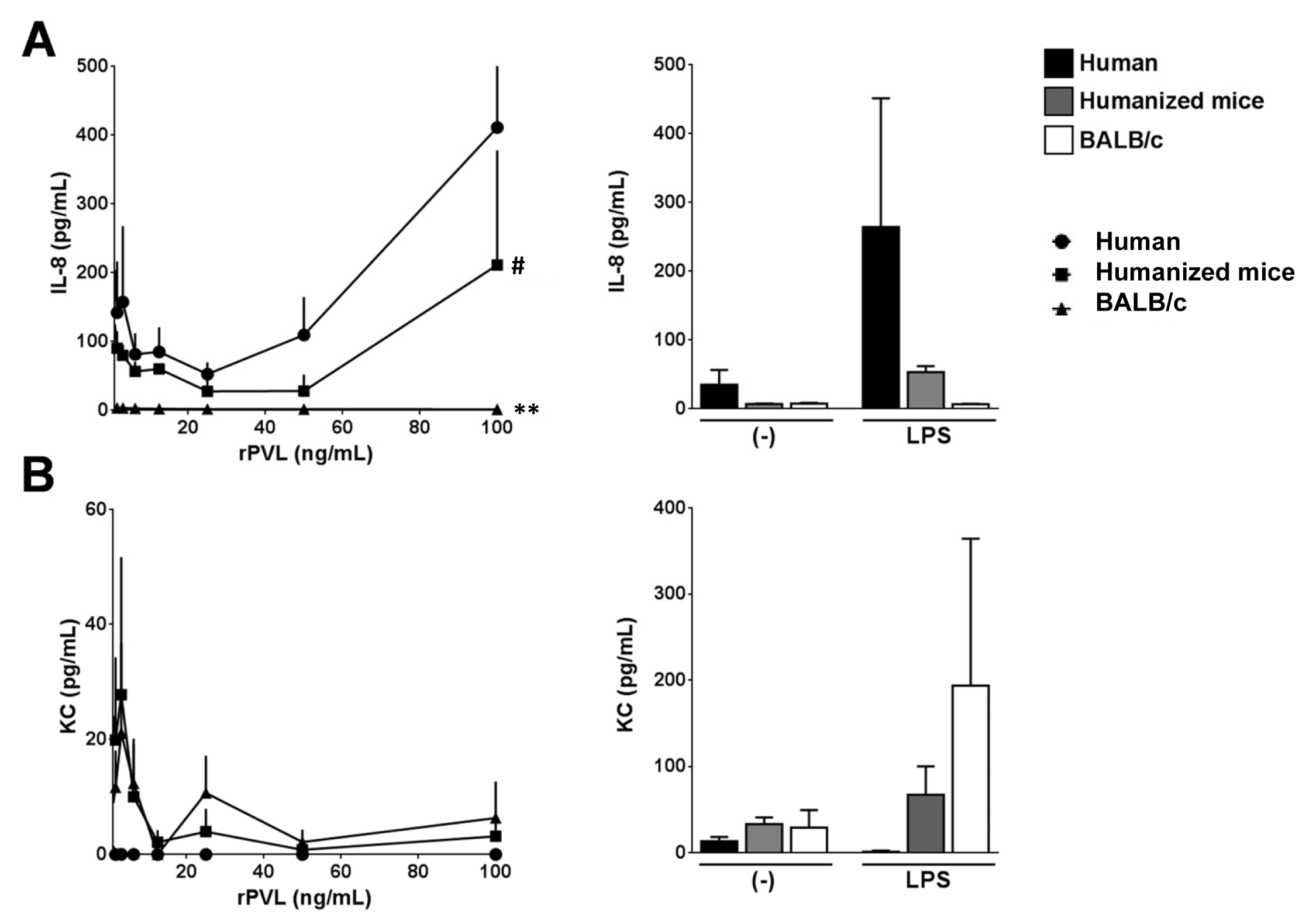

Supplement: S3 Fig — PMN were isolated from the blood of human volunteers and bone marrow of humanized mice or BALB/c mice. The PMN preparations were incubated with rPVL. After 3 h, (A) human IL-8 (n = 3, p < 0.05, two-way ANOVA) and (B) mouse KC (n = 3, p < 0.01, two-way ANOVA) were measured from the PMN supernatants. Human IL-8 and murine KC levels following PMN incubation with medium or 100 ng/mL LPS are also shown. **: p <0.01 compared to human; #: p < 0.05 compared to mouse (n = 3 experiments). (TIF) [file ppat.1005292.s004.tif]

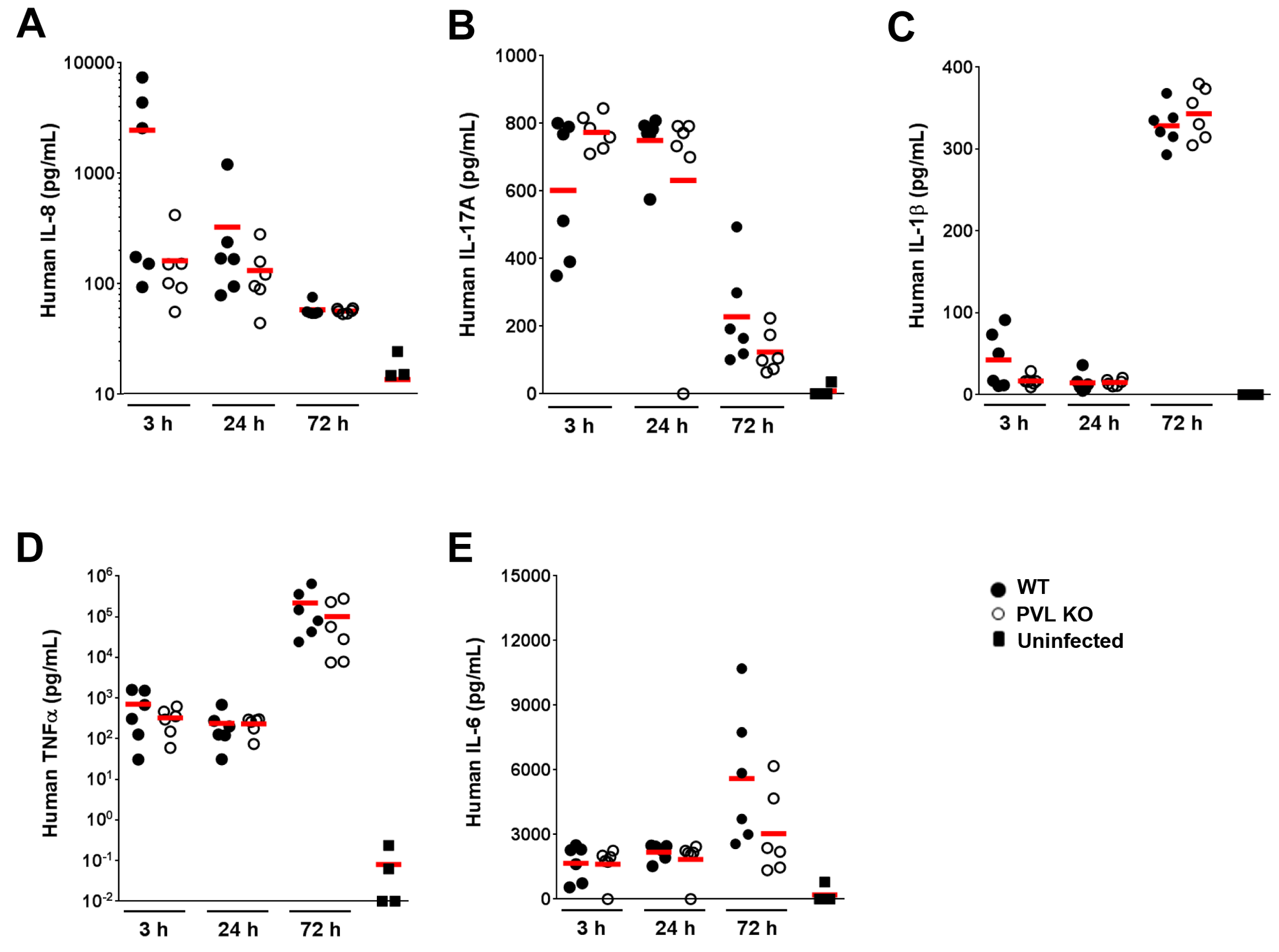

Supplement: S4 Fig — Humanized mice were infected on the left flank with 106 CFU WT S. aureus and on the right flank with 106 CFU PVL- isogenic mutant strain. The mice (n = 4–6 mice per group) were sacrificed at various time points post-infection. Shown are (A) Human IL-8, (B) IL-17A, (C) IL-1β, (D) TNF-α, and (E) IL-6 from the infection sites. UI: uninfected. Red bar = mean. (TIF) [file ppat.1005292.s005.tif]

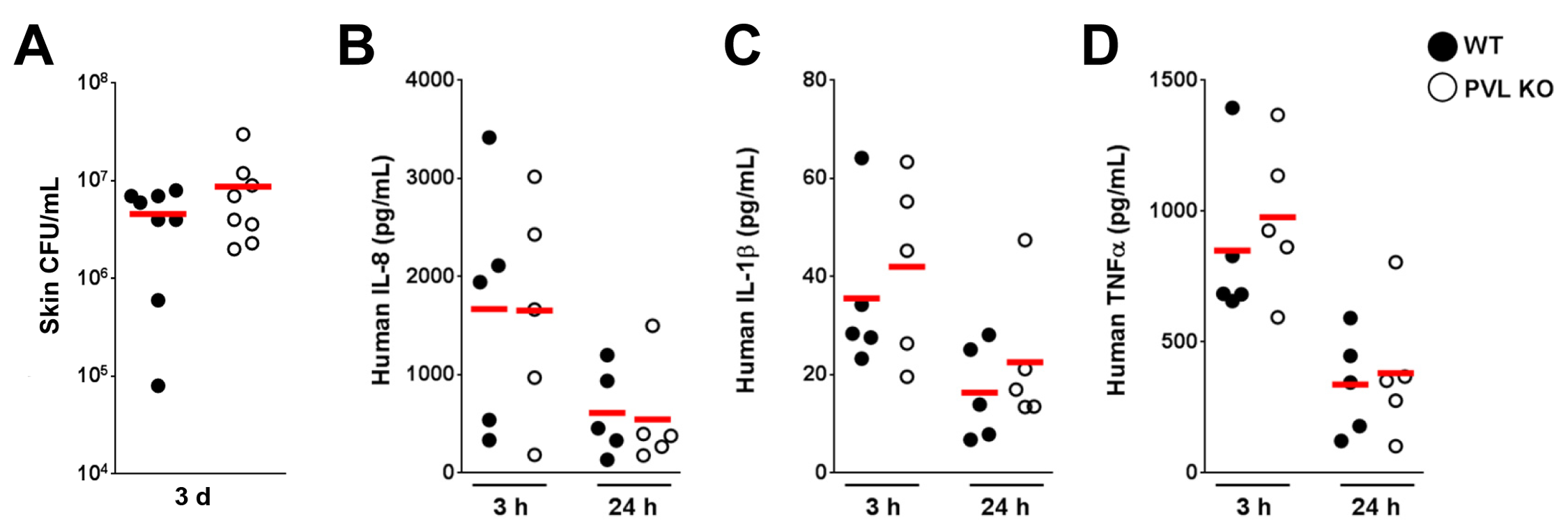

Supplement: S5 Fig — NSG mice were injected i.v. with 5 x 10^6^ human PMN. Three hours later, the mice were infected on the left flank with 106 CFU WT S. aureus and on the right flank with 106 CFU PVL- isogenic S. aureus, and sacrificed 3 h or 24 h after infection. Shown are (A) bacterial CFU, (B) human IL-8, (C) IL-1β, and (D) TNFα from the infection sites. Red bar = mean. (TIF) [file ppat.1005292.s006.tif]

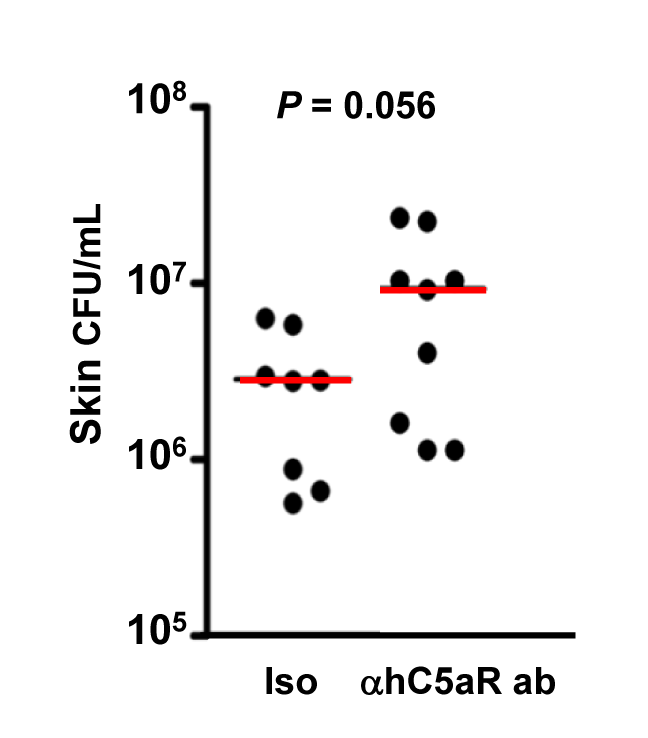

Supplement: S6 Fig — Humanized NSG mice were infected on the left flank with ~2 x 10^6^ WT S. aureus and on the right flank with ~2 x 10^6^ CFU PVL- isogenic S. aureus. After 3 h, the mice were injected i.p. with anti-hC5aR or an isotype control antibody (8 mg/kg/d). Bacterial burden was measured on d 3. Red bar = mean. (TIF) [file ppat.1005292.s007.tif]
